# Activating transcription factor 4: a regulator of stress response in human cancers

**DOI:** 10.3389/fcell.2024.1370012

**Published:** 2024-03-27

**Authors:** Di Wu, Jie Liang

**Affiliations:** Department of Neurosurgery, The Fourth Affiliated Hospital of China Medical University, Shenyang, Liaoning, China

**Keywords:** stress, metabolism, cancer, transcription, autophagy

## Abstract

Activating transcription factor 4 (ATF4) is an adaptive response regulator of metabolic and oxidative homeostasis. In response to cellular stress, ATF4 is activated and functions as a regulator to promote cell adaptation for survival. As a transcriptional regulator, ATF4 also widely participates in the regulation of amino acid metabolism, autophagy, redox homeostasis and endoplasmic reticulum stress. Moreover, ATF4 is associated with the initiation and progression of glioblastoma, hepatocellular carcinoma, colorectal cancer, gastric cancer, breast cancer, prostate cancer and lung cancer. This review primarily aims to elucidate the functions of ATF4 and its role in multiple cancer contexts. This review proposes potential therapeutic targets for clinical intervention.

## Introduction

Tumor cells are accompanied by the metabolic stress in tumor microenvironment due to the accelerated growth (Payne, 2022). Poor oxygen and deprived nutrient supply elicit metabolic stress, making tumor cells reprogrammed for adaptive mechanisms. Tumor cells can initiate cellular adaptation to rewire their metabolic phenotype to cope with these metabolic stresses (Jin and White, 2007). Targeting these cellular adaptations may provide potential ways for anti-tumor strategy.

**FIGURE 1:**
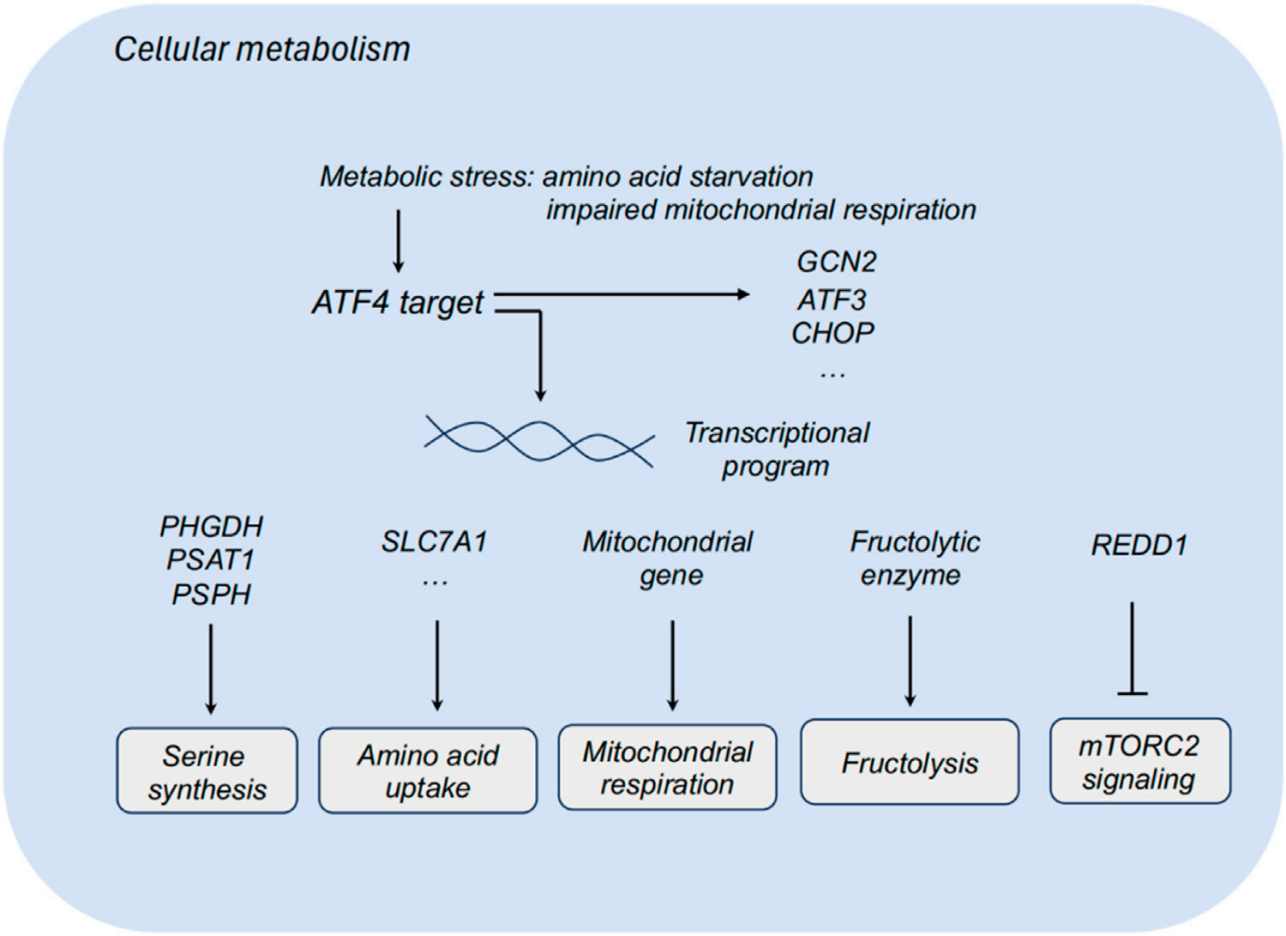
ATF4 and cellular metabolism. ATF3, activating transcription factor 3; ATF4, activating transcription factor 4; CHOP, C/EBP homologous protein; PHGDH, phosphoglycerate dehydrogenase; PSAT1, phosphoserine aminotransferase 1; PSPH, phosphoserine phosphatase; SLC7A1, solute carrier family 7 member 1; REDD1, regulated in development and DNA damage response.

**FIGURE 2:**
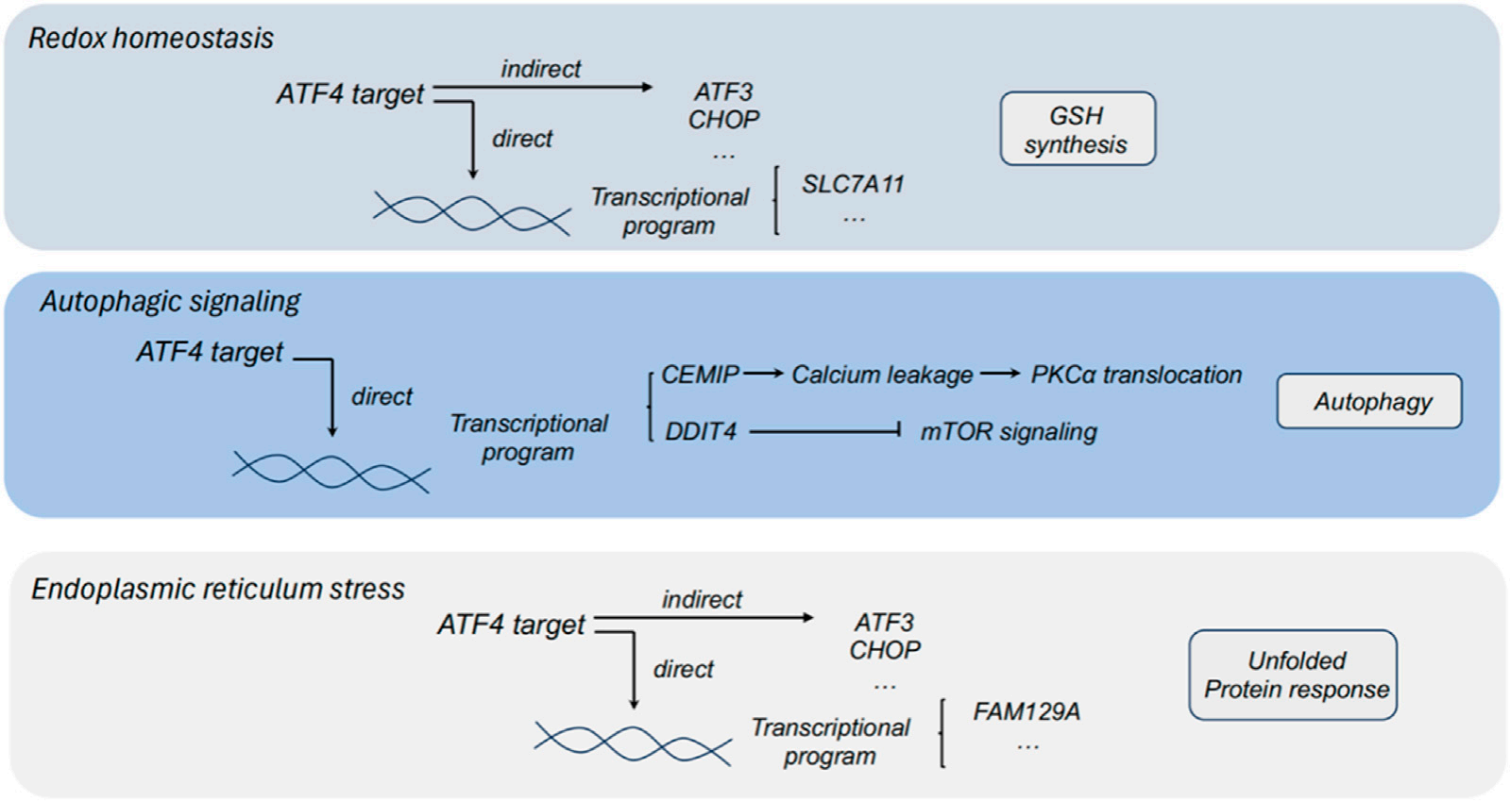
The role of ATF4 in redox homeostasis, autophagic signaling and endoplasmic reticulum stress. ATF3, activating transcription factor 3; ATF4, activating transcription factor 4; CHOP, C/EBP homologous protein; PHGDH, phosphoglycerate dehydrogenase; PSAT1, phosphoserine aminotransferase 1; PSPH, phosphoserine phosphatase; SLC7A11, solute carrier family 1 member 11; GSH, glutathione; DDIT4, DNA damage inducible transcript 4; CEMIP, cell migration inducing protein; FAM129A, family with sequence similarity 129 member A.

In response to diverse cellular and metabolic stress, Activating transcription factor 4 (ATF4) is elevated and acts as a regulator to promote cell adaptation for survival (Wortel et al., 2017). In cancers, ATF4 has been identified as a stress-induced transcription factor and found to be frequently upregulated in a series of tumors. Notably, ATF4 has been detected to highly express in some hypoxic and nutrient-poor tumoral regions (Ye and Koumenis, 2009). As a transcriptional regulator, ATF4 widely participates in the regulation of amino acid metabolism, autophagy, redox homeostasis and endoplasmic reticulum stress in tumors (Figures 1, 2). Herein, we comprehensively summarized the diverse role of ATF4 in tumors, exploring the clinical implications of targeting ATF4 for anti-tumor strategy (Table 1).

**TABLE 1:** Role of ATF4 in human cancer.

*Cancer type*	*Role of ATF4 in cancer*
*Glioblastoma*	*Proliferation, stemness and angiogenesis*
*Hepatocellular carcinoma*	*Proliferation, stemness and sorafenib resistance*
*Colorectal cancer*	*Proliferation*
*Gastric cancer*	*Proliferation, invasion and chemoresistance*
*Breast cancer*	*Proliferation and radio-resistance*
*Prostate cancer*	*Anoikis resistance, resistance to androgen-deprivation therapy*
*Lung cancer*	*Proliferation and invasion*

## ATF4 structure and regulation

**FIGURE 3:**
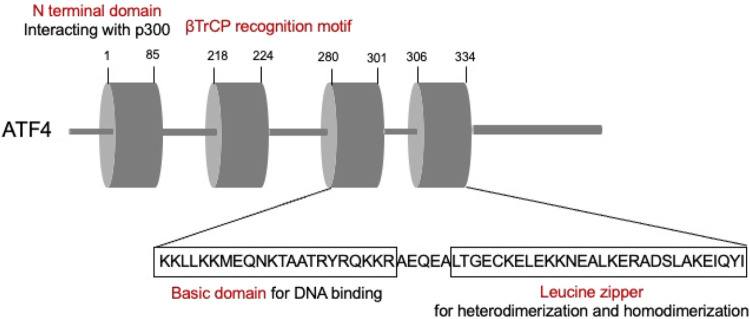
Schematic illustrating ATF4 structure.

ATFs are comprised by basic region-leucine zipper (bZip) proteins, which could interact with other bZIPs-containing proteins (Hai and Hartman, 2001). ATFs are primarily comprised by two parts. The basic element is the continuous sequence “TGACGTCA,” which could bind to DNA, while the leucine zipper mediates heterodimerization and homodimerization (Ameri and Harris, 2008). Among these, ATF4, the critical member of the ATF family, is an adaptive response regulator of metabolic and oxidative homeostasis (Quirós et al., 2017) (Figure 3). ATF4 contact with DNA sequences called cAMP responsive elements (CREs) and corporately regulate the expression of the target genes.

In response to diverse stress, cancer cells could activate an adaptive program to sustain metabolic and oxidative homeostasis. The key event is the phosphorylation of the α subunit of the eukaryotic initiation factor eIF2 (eIF2α), leading to the activation of transcription factor ATF4 (Pakos-Zebrucka et al., 2016). The eIF2α/ATF4 axis activates transcriptional program in response to different cellular stress (B’chir et al., 2013). PKR-like ER kinase (PERK), general control non-derepressible 2 (GCN2), protein kinase RNA-activated (PKR) and heme-regulated eIF2α kinase (HRI) are main kinases to sense the cellular stress and mediate the phosphorylation of eIF2α. In addition, MYC could induce the activation of ATF4 by GCN2 through uncharged transfer RNAs (Tameire et al., 2019). ATF4 has been identified as a metabolic effector of mTORC1 to regulate the expression of genes involved in cystine uptake and glutathione synthesis (Torrence et al., 2021).

## ATF4 as a metabolic regulator

Tumor cells reprogram their metabolism to promote cell survival and proliferation in nutrient-deprived environment. Non-transformed cells exhibit relatively low demands for serine and glycine, while some tumor cells display high demands for serine and glycine biosynthesis. Dependent on these biosynthetic pathways, tumor cells could promote macromolecule biosynthesis and eliminate oxidative stress. Multiple metabolic enzymes of serine synthesis pathways, including phosphoglycerate dehydrogenase (PHGDH), phosphoserine aminotransferase 1 (PSAT1) and phosphoserine phosphatase (PSPH), participates in the serine synthesis, which sustains tumor cells to survive and proliferate in serine-limiting conditions. Upon serine deprivation, ATF3 is activated relying on ATF4 to further interact with ATF4 and sustain its protein stability, thereby promoting serine synthesis via a positive feedback loop (Li X. et al., 2021). Targeting PHGDH, the first step in the serine biosynthesis, combined with serine starvation blocks the activation of an ATF4-mediated gene program (Tajan et al., 2021). ATF4 also functions as a metabolic effector of mTORC1 in regulating cystine uptake and glutathione biosynthesis (Torrence et al., 2021).

Impaired mitochondrial respiration leads to the activation of ATF4, and ATF4 could upregulate the expression of mitochondrial-related genes and relieving mitochondrial stress. In the context of impaired mitochondrial respiration, asparagine could rescue ATF4 activation, and ATF4 promotes the transcription of genes in mitochondrial respiration and stress relieving to produce asparagine precursors for asparagine synthesis. Asparagine acts as a signal for mitochondrial respiration to ATF4, and that asparagine deprivation leads to impaired proliferation with inhibited respiration (Krall et al., 2021).

## The role of ATF4 in cancers

### Glioblastoma

Glioblastoma is the most common intracranial malignancy and constitutes about 50% of all gliomas (Wang Y. et al., 2023; Schaff and Mellinghoff, 2023). Biological properties of ATF4 in glioblastoma have been extensively documented in a series of studies. It has been found that ATF4 plays a key role in proliferation, stemness and drug resistance of glioblastoma cells via complex mechanisms.

It has been found that glioblastoma cells could shift their energetic demand from glycolysis to fructolysis in response to glucose limitation. Mechanically, glucose limitation could drive upregulation of fructolytic enzymes by specifically enhancing translation of ATF4 in U87 and LN229 cells. ATF4 inhibition impaired fructolysis to suppress growth of glioblastoma cells. Herein, ATF4-mediated fructolysis is an essential metabolic adaptation, indicating a promising target for the treatment of glioblastoma (Chen et al., 2022).

Tumor-initiating cells (TICs) is critical for heterogeneity and relapse of glioblastoma, making TIC cells potential targets for the eradication of glioblastoma (Gimple et al., 2019). In patient-derived xenograft models of glioblastoma, lysine specific demethylase 1 (LSD1) is vital for maintaining growth and self-renewal of TICs. ATF4 has been identified as a pivotal regulator of the integrated stress response. LSD1 ablation could inhibit ATF4 activation, resulting in aberrant integrated stress response and sensitizing TICs to stress-induced cell death, ultimately suppressing tumor progression. Functionally, LSD1 ablation could disturb LSD1 scaffolding capability and inhibit its interaction with CREBBP, a key ATF4 activator (Faletti et al., 2021). The complex interaction between LSD1 and ATF4 in maintaining formation of glioblastoma multiforme is critical for the transcriptional response of TICs to stress.

In addition, glutamate antiporter xCT/SCL7A11 has been identified as a downstream target of ATF4 in gliomas. xCT, importing cystine for the biosynthesis of the glutathione, which mediates tumor malignancy and chemoresistance (Koppula et al., 2021). Also, xCT is critical for cell ferroptosis, a form of iron-related cell death. ATF4 could promote tumor angiogenesis and shape the vascular architecture in a xCT-dependent manner. ATF4 promoted glioma cell proliferation and glutamate secretion through xCT regulation in U87 and U251 cells. The promoting effect of ATF4 on ferroptosis-mediated cell death and tumor angiogenesis can be attenuated by xCT inhibition. Furthermore, ATF4-mediated angiogenesis could be abrogated by erastin. Thus, ATF4 ablation is a promising target for suppressing growth and vasculature of gliomas by sensitizing glioma cells to ferroptosis (Chen et al., 2017).

Glioblastoma is commonly characterized by dysregulated vascularity that supports the formation of a hypoxic and immune-hostile vascular microenvironment and induces resistance to T cell-based immunotherapy. In glioblastoma, PHGDH has been identified as a key target of ATF4 in endothelial cell (EC) metabolism (Zhang et al., 2023). Signals from TME leads to ATF4-induced PHGDH upregulation in ECs, a redox-dependent mechanism that regulates endothelial glycolysis and promotes EC growth. Metabolic adaptation of ECs by targeting ATF4-PHGDH axis provide a promising strategy to improve the efficacy of T cell-based immunotherapy.

Loperamide (LOP) is an autophagic cell death-inducing drug. Recent study has shown a novel ATF4-mediated mechanisms that underlie the cytotoxic effect of LOP. In glioblastoma cells. LOP could upregulate ATF4 expression, accompanied by multiple ER stress markers. ATF4 ablation suppressed LOP-induced autophagy and autophagic cell death (Zielke et al., 2021). Experimental study revealed Withaferin A (WA), a bioactive compound with anti-tumor effect derived from Withania somnifera, induced ER stress through the ATF4-ATF3-CHOP axis to promote cell apoptosis and G2/M arrest in glioblastoma cells (Tang Q. et al., 2020). In a word, ATF4 could modulate metabolic reprogramming, integrated stress response and ferroptosis to promote progression of glioblastoma. Collectively, ATF4 is a potential target for glioblastoma.

### Hepatocellular carcinoma

Hepatocellular carcinoma (HCC) is a primary liver malignancy commonly developed from cirrhosis and some chronic liver diseases (Hartke et al., 2017). In late-stage HCC, sorafenib is currently an effective first-line therapy (Tang W. et al., 2020). ATF4 has been identified as a driver of resistance to sorafenib in HCC by preventing ferroptosis. Mechanistically, YAP/TAZ could stabilize ATF4 protein and sustains nuclear localization of ATF4, which corporately regulated SLC7A11 expression to promote sorafenib resistance (Gao et al., 2021). In sorafenib-resistant HLE cells, ATF4 mediated the upregulation of SLC7A11 expression to elevate glutathione (GSH) levels, reduce ROS levels and inhibit ferroptosis, making HCC cells survive in the presence of sorafenib. In normal hepatocytes, ATF4 plays an important protective role in hepatosteatosis. However, ATF4 ablation could increase susceptibility to ferroptosis and promote HCC development. ATF4 directly target SLC7A11 to sustain GSH synthesis and reduce ferroptosis-dependent cell death (He et al., 2023). Moreover, ferroptosis inhibitors or ATF4 activators may impair the tumorigenesis of HCC. In addition, ATF4 could directly bind to the promoter of fucosyltransferase 1 (FUT1) to promote drive FUT1 transcription for HCC development in Huh7 and CLC13 cells (Loong et al., 2021). Glucose restriction activates the PERK/eukaryotic initiation factor 2α (eIF2α)/ATF4 axis to promote FUT1 transcription. FUT1 mediates the fucosylation of downstream targets, leading to deregulated AKT/mTOR/4EBP1 signaling for tumor stemness. Notably, α-(1,2)-fucosylation inhibitor could sensitize HCC tumors to sorafenib and impair tumorigenesis. Taken together, ATF4 may be exploited as an effective target for HCC.

### Colorectal cancer

Colorectal cancer (CRC) is one of the most commonly diagnosed cancer (Siegel et al., 2023). CRC cells often require glutamine to support tumor survival and growth (Cluntun et al., 2017). ATF4 could activate glutamate pyruvate transaminase 2 (GPT2) transcription in PIK3CA mutant HCC cells, making them rely on glutamine. Functionally, mutant p110α could drive PDK1-RSK2 axis to activate ATF4. Phosphorylation of ATF4 S245 by RSK2 could enhance its binding to USP8 and enhance the protein stability of ATF4. Thus, targeting glutamine metabolism is a promising strategy for CRC patients with PIK3CA mutations (Hao et al., 2016).

Enhanced glutaminolysis could provide energetic advantages to CRC cells, making inhibition of glutaminolysis as a potential anti-cancer strategy. Upon glutaminolysis inhibition, ATF4-mediated pathway has been found to be activated in CRC cells. Glutaminolysis inhibition could activate ATF4 by attenuating N6-methyladenosine (m6A) modification and YTHDF2-mediated RNA decay. Moreover, ATF4 could upregulate DNA damage inducible transcript 4 (DDIT4) to inhibit mTOR signaling for pro-survival autophagy during glutaminolysis inhibition. Targeting ATF4-mediated autophagic program is a potential strategy to combine with glutaminolysis-inhibition for CRC treatment (Han et al., 2021).

### Gastric cancer

Gastric cancer remains to rank fifth for incidence and fourth for mortality globally (Siegel et al., 2023). With the progress of early diagnosis and comprehensive treatment strategies for gastric cancer, the therapeutic effect of gastric cancer has been greatly improved. Unfortunately, the total 5-year overall survival of advanced GC is still poor. Therefore, identification of promising therapeutic targets for GC is extremely urgent to improve the prognosis of patients with GC.

Multiple studies have reported that the mRNA and protein expression of ATF4 are markedly upregulated in GC tissues than para-cancerous tissues. ATF has been found to participate in GC initiation and progression via various mechanism. ATF4 could bind to the promoter of Sonic Hedgehog (SHH) to activate the SHH pathway, and ATF4 could promote proliferation and invasion of GC cells through activating SHH pathway (Wang L. M. et al., 2023). ATF4 acts a pivotal transcription factor in nucleotide synthesis. U2AF homology motif kinase 1 (UHMK1) directly phosphorylates NCOA3 at S1062 and T1067 to enhance its binding to ATF4 and upregulate the expression of purine metabolism-related genes. Moreover, ATF4 directly activates the transcription of the UHMK1 promoter in GC, forming a positive loop (Feng et al., 2020). ATF4 also upregulates the expression of ChaC glutathione-specific γ glutamylcyclotransferase 1 (CHAC1) to degrade GSH and mediate ferroptosis. Chemoresistance remains a major obstacle for advanced GC. It has been demonstrated that ATF4 is downregulated in chemo-resistant GC cells. ATF4 overexpression could reverse chemoresistance by activating CHOP to promote cell apoptosis (Feng et al., 2021). Moreover, casein kinase 1 delta (CK1δ) participates in the ATF4-S219 phosphorylation, further promoting proteasomal degradation of ATF4 (Feng et al., 2021). The complex role of ATF4 on oncogenic signaling, purine metabolism, ferroptosis and chemoresistance indicates ATF4 is a promising therapeutic target for GC.

### Breast cancer

Currently, breast cancer has surpassed lung cancer to be the most commonly diagnosed cancer globally. According to the expression of hormone receptors, human epidermal growth factor receptor 2 (HER2) and Ki-67, the intrinsic molecular subtypes of breast cancer have been defined and characterized as HER2-enriched, luminal A, luminal B and triple-negative breast cancer (TNBC). With the advances in early diagnosis and treatment of breast cancer, the 5-year survival rate of breast cancer has been significantly improved, nearly reaching 80% (Barzaman et al., 2020). However, the recurrence and metastasis of breast cancer are still threatening the life of breast cancer patients.

ATF4 has been found to play a key role in occurrence and progression of breast cancer. PSAT1 is a critical downstream target of ATF4. ATF4 can directly activate PSAT1 to enhance cell proliferation via the GSK3β/β-catenin/cyclin D1 signaling pathway in ER-negative breast cancer (Gao et al., 2017). Additionally, ATF4 mediated radio-resistance by activating integrated stress response in TNBC cells. In radioresistant MDA-MB-231 and MDA-MB-436 cells, eIF2α phosphorylation facilitated ATF4 activation and induced the transcription of downstream genes involved in GSH biosynthesis to elevate the intracellular level of reduced GSH and the scavenging of ROS after irradiation, thereby leading to a radio-resistant phenotype. Instead, eIF2α dephosphorylation increased irradiation-mediated ROS accumulation in radio-resistant TNBC cells by disrupting ATF4-mediated GSH biosynthesis. The eIF2α/ATF4-mediated ROS elimination uncovers a key mechanism underlying TNBC radio-resistance, which may elicit novel therapeutic targets for TNBC treatment (Bai et al., 2021). ATF4 also mediated resistance of breast cancer cells treated with bortezomib by upregulating LC3B. Bortezomib is a selective inhibitor of the 26S proteasome and is approved for the treatment of multiple myeloma. Clinical trials with bortezomib have shown promising results for breast cancer. Bortezomib can induce the unfolded protein response and autophagy in MCF-7 breast cancer cells. Autophagy induced by bortezomib depends on the stabilization of ATF4 and upregulation of LC3B mediated by ATF4. Thus, ATF4 is essential for protecting cells from bortezomib-induced cell death (Milani et al., 2009).

### Prostate cancer

Prostate cancer is one of the primary causes of cancer-related death in men globally. ATF4 has been found to be critical for survival and growth of prostate cancer. Family with sequence similarity 129 member A (FAM129A) has been identified as a direct target of ATF4, mediating tumorigenic effects of ATF4. Mechanistically, FAM129A could regulate PERK-eIF2α axis to channel the output of unfolded protein response (Pällmann et al., 2019). Besides, ATF4 could directly bind to cell migration inducing protein (CEMIP) 3′UTR to enhance CEMIP transcription. CEMIP further promotes calcium leakage from the endoplasmic reticulum to induce Protein kinase C (PKC) α translocation to enhance autophagy via Bcl-2/Beclin1 complex dissociation, leading to anoikis resistance of prostate cancer (Yu et al., 2022).

Despite the development of androgen-deprivation therapy (ADT), some patients with metastatic prostate cancer may finally progress to castration-resistant prostate cancer (CRPC) (Mansinho et al., 2018; Teo et al., 2019). Therapeutic strategies are desperately needed for treating metastatic CRPC that is unresponsive to ADT and chemotherapy. PKCλ/ι is downregulated and loss of PKCλ/ι plays a vital role in promoting tumor development of CRPC. ATF4 functions as the main upstream regulator of the transcriptional alterations occurred in PKCλ/ι-deleted cells. PKCλ/ι loss could activate mTORC1 to induce an ATF4-mediated transcriptional program, leading to the metabolic adaptation of CRPC cells to elevate the flux through the one-carbon pathway (Reina-Campos et al., 2019). Thioesterase superfamily member 6 (THEM6) has been identified as a marker of ADT resistance. THEM6 loss in CRPC cells could reprogram endoplasmic reticulum status, inhibiting sterol biosynthesis and ATF4 activation (Blomme et al., 2022).

### Lung cancer

Lung cancer is the primary cause of cancer-related death. Moreover, lung cancer is often diagnosed at a relatively advanced stage with a poor prognosis (Mao et al., 2016). ATF4 regulates metabolic adaptation, tumor dormancy and autophagy of lung tumor cells in complex manners. Overexpression of ATF4 has been found to significantly promote tumor cell growth and invasion (Du et al., 2021).

Under metabolic stress like amino acid limitation, ATF4 functions as a critical regulator for adaptive program. ATF4 could activate the expression of genes involved in serine biosynthesis in response to serine starvation in lung cancer cells. NRF2 activates the ATF4-induced expression program of PHGDH, PSAT1 and SHMT2 in A549 cells (DeNicola et al., 2015). These enzymes mediate the production of serine and glycine from the glycolytic intermediate 3-PG and funnel the carbon into glutathione and nucleotides via the folate and trans-sulfuration cycles. Upon amino acid starvation, general control nonderepressible 2 (GCN2) kinase senses intracellular amino acid levels and thereby activates ATF4 expression to induce regulated in development and DNA damage response (REDD1) expression. In H1299 cell, ATF4 silencing could block REDD1 expression and further lead to mTORC2-induced AKT activation upon amino acid deprivation, indicating the pivotal role of ATF4 in REDD1 expression and mTORC2-induced AKT activation for cell survival under amino acid starvation (Jin et al., 2021).

Slow-cycling/dormant cancer cells (SCCs) display a dormancy-like phenotype and pro-survival potential, leading to tumor relapse and resistance (Phan and Croucher, 2020). The key regulator G protein signaling 2 (RGS2) could endows SCCs dormancy-like phenotypes and pro-survival ability against endoplasmic reticulum stress by interfering ATF4-mediated cellular program. In SCCs, RGS2 could bind with ATF4 by the N-terminal residues of RGS for ATF4-induced proteasomal degradation, therefore extending translational arrest. This RGS2/ATF4 axis is a potential target to reduce tumor relapse and resistance of NSCLC patients (Cho et al., 2021). Autophagy, the degradation of impaired organelles and misfolded proteins, is critical for tumor cell survival. Upon stress conditions, the eIF2α-ATF4 axis is essential for mediating cell autophagy in non-small cell lung cancer (NSCLC). TOR signaling pathway regulator-like (TIPRL) could interact with eIF2α and promote eIF2α phosphorylation to activate the eIF2α-ATF4 axis, thereby promoting transcription of autophagy-related genes and enhancing autophagy (Jeon et al., 2019). The eIF2α-ATF4 axis in controlling autophagy induction may be exploited as a potential strategy for the anti-NSCLC therapies.

### Conclusion

In brief, ATF4 serves as an adaptive response regulator of metabolic and oxidative homeostasis. The complex role of ATF4 in the regulation of amino acid metabolism, autophagy, redox homeostasis and endoplasmic reticulum stress is essential for balancing the pro- and anti-survival signals of ATF4. Collectively, exploring the underlying mechanisms of ATF4-induced transcriptional program and its effects on cellular adaptation may provoke novel therapeutic target for anti-tumor strategy.
